# Direct Visualization of Perm-Selective Ion Transportation

**DOI:** 10.1038/s41598-020-65433-y

**Published:** 2020-06-01

**Authors:** Wonseok Kim, Jungeun Lee, Gunsu Yun, Gun Yong Sung, Sung Jae Kim

**Affiliations:** 1 Department of Electrical and Computer Engineering, Seoul National University, Seoul, 08826 Republic of Korea; 2 Department of Bioengineering, University of California, Berkeley, CA 94720 USA; 3 Department of Material Science and Engineering, Hallym University, Chuncheon, 24252 South Korea; 4 Department of Physics, Pohang University of Science and Technology, Pohang, 37673 South Korea; 5 Nano System Institute, Seoul National University, Seoul, 08826 South Korea; 6 Inter-university Semiconductor Research Center, Seoul National University, Seoul, 08826 South Korea

**Keywords:** Nanofluidics, Fluid dynamics

## Abstract

Perm-selective ion transportation in a nanoscale structure such as nanochannel, nanoporous membrane or nanojunction has been extensively studied with aids of nanofabrication technology for a decade. While theoretical and experimental advances pushed the phenomenon to seminal innovative applications, its basic observation has relied only on an indirect analysis such as current-voltage relation or fluorescent imaging adjacent to the nanostructures. Here we experimentally, for the first time, demonstrated a direct visualization of perm-selective ion transportation through the nanoscale space using an ionic plasma generation. A micro/nanofluidic device was employed for a micro bubble formation, plasma negation and penetration of the plasma along the nanojunction. The direct observation provided a keen evidence of perm-selectivity, *i*.*e*. allowing cationic species and rejecting anionic species. Furthermore, we can capture the plasma of lithium, which has lower mobility than sodium in aqueous state, passed the nanojunction faster than sodium due to the absence of hydrated shells around lithium. This simple, but essential visualization technique would be effective means not only for advancing the fundamental nanoscale electrokinetic study as well as interfacial ion transportation between liquid and plasma but also for providing the insight of new innovative engineering applications.

## Introduction

Ion selective transportation through nanoporous membrane (or nanochannel) has been extensively studied due to its fundamental importance to understand basic biological functions^1–3^ and developing innovative engineering applications such as electro-desalination^4–6^ and high energy efficient battery^7–9^. Especially, single ion selective pump has been drawn ever-increasing attentions, for example, potassium selective pump and sodium selective pump in cell membrane for physiological homeostasis of living organism^10,11^ or high voltage generation of electrical eel^12^. More recently, extracting lithium ions out of abundant sodium ions in seawater has become key issues in lithium ion battery industry for supplying economic raw material^13,14^. In addition, perm-selectivity in ion-selective sensors is also extremely important. In these sensors, perm-selectivity is typically achieved by adding anionic or cationic site to a hydrophobic phase. Such a strategy has been utilized in applications with various electrodes and optodes for ions^15–17^. Those high industrial demands should require a proper understanding of nanostructure and its fundamental interaction with adjacent electrolyte solution.

Engineered nanostructures has a characteristic length scale less than electrical double layer of few nanometers formed adjacent to the interface of electrolyte/solid^18^. Thus, electrical polarity inside a confined nanostructure should be either positive or negative depending on the surface charge of solid substrate. If the solid such as silicon and glass is negatively charged, only cations can pass through the nanoscale space between these solids, while anions are rejected to enter the nanoscale space. This is conventional working principle of perm-selectivity^19^. While several works successfully demonstrated the working principles, major nuisance is that their investigations typically have relied on indirect methods such as measuring current-voltage relation^20–22^, (fluorescent) imaging^23–25^ or analyzing a transported sample fluid by mass spectrometry^26,27^, *etc*., mainly because direct and *in situ* visualization inside nanoscale space is highly challenging task. Compared to perm-selective ion transportation, an ion transportation can be visualized even with a simple litmus solution. Migration of H^+^ through a nanopore can be visualized by the color change of litmus solution in proton-receiving side of the nanopore^28,29^. Thus, profound debates are still ongoing for the origin of ion selectivity and its related phenomena that has never been demonstrated^18,30–35^. In this sense, the direct visualization of perm-selective ion transportation through nanostructure becomes priceless subject to be investigated.

On the other hand, studies related to the plasma generation at the atmospheric pressure and the low temperature condition in the microfluidic device have been reported^36,37^, but there have been few studies on the utility of inherent luminescence characteristics due to the obvious disadvantages such as high voltage operation and the instability^38,39^. In the generated spark plasma, both atoms and ions can co-exist via various ionizations and charge recombination processes with electrons^40^. For example, chlorine atoms coexist with chlorine ions via dissociative excitation of Cl_2_^41^; sodium atoms coexist with sodium ions via ionization and charge recombination processes. Therefore, from the plasma chemistry point of view, the presence of these atoms is possible in the nanoscale space that our experiments were conducted. More importantly, similar to the concept of electrical double layer overlap in nanoelectrokinetic research field, the Debye sheath layer(electrically non-neutral layer) which forms between plasma and solid substrate^41,42^ is much thicker (approximately 10 μm for the experimental conditions in this work of room temperature, 1 atm and surface charge of Nafion as −200 ~ −600 mC/m^2^ ^43,44^) than the dimension of the nanochannel. This implies that the charge-neutrality condition is broken inside the nanoscale space between the substrates and a proton rich region is formed inside the nanoscale space. Thus, strongly electronegative chlorine atoms, which can become excited and radiate in violet spectrum in the plasma^45^, easily combined with electrons within the Debye sheath layer (Note that the electron affinity of chlorine atom (2.45 eV) is comparable to the dissociation energy of Cl_2_ (2.5 eV)^41^ and subsequently chlorine ions are rejected from entering the nanoscale space. This is why the perm-selectivity is retained inside the nanoscale space. Here, we employed this plasma discharge inside a micro/nanofluidic platform that enables to generate plasma in stable manner for reporting the direct observation of perm-selective transportation for the first time.

## Methods

### Device fabrication

The microchannels were molded by the general PDMS fabrication process^46^. Briefly, PDMS solution at the ratios of pre-polymer (PDMS, Sylgard 184, Dow corning) to curing agent of 10:1 was mixed and degassed for an hour. After pouring on lithographically constructed Si wafer, it was cured at 75 °C for 4 hours. The demolded PDMS block and Nafion (Sigma Aldrich, USA)-patterned glass substrate were irreversibly adhered by O_2_ plasma treatment (Cute-MP, FemtoScience, Korea). The Nafion was patterned by surface patterned method^47,48^. Both microchannels were filled with 2 M NaCl or 2 M LiCl solution.

**Figure 1:**
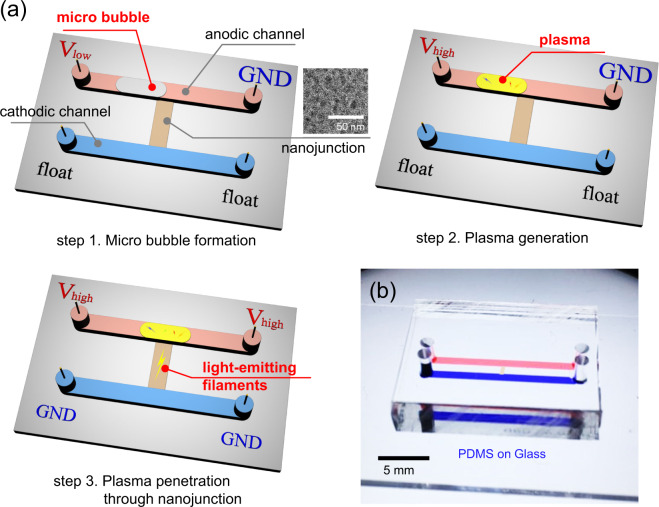
(**a**) Schematic diagrams of micro/nanofluidic platform for the direct visualization of perm-selective ion transportation using an ionic plasma generation. Experimental steps were (i) micro bubble formation inside a microchannel, (ii) plasma generation in the microchannel and (iii) plasma penetration through a nanojunction. (**b**) Fabricated micro/nanofluidic device.

 Nafion nanojunction has a number of O(10) nm nanopores inside the structure^43^ as shown in the inset (TEM image) in Fig. 1(a). Simply, it could be thought as a rectangular sponge of 100 μm in width, 5 mm in height, and 1.5–1.8 μm in depth. If more diluted Nafion resin is used or the depth of blank microchannel is changed, the depth of nanojunction can be adjusted to 100 nm–1 μm if necessary. The reasons for the use of Nafion junction are (i) high surface charge of Nafion to obtain high perm-selectivity at physiological electrolyte concentration over 100 mM^5,49^ and (ii) easiness of fabrication^50^. A nanochannel lithographically made of silicon, glass or PDMS lost its perm-selectivity at an electrolyte concentration over 100 mM due to its thin electrical double layer. Also, Nafion can be easily patterned on a slide glass even at a regular laboratory^47,50,51^, and this nanojunction fabrication does not require any nano-lithographical facility.

For a transmittance experiment, Nafion solution was spun-coated on microscopic slide glass at 4,000 rpm, which provided 10 ± 2 μm thick Nafion film. For a wet film, 2 M NaCl was dropped on top of the film.

### Experimental setups

Ag/AgCl electrodes were inserted at both reservoirs to apply external voltage (PS 350, Stanford Research Systems, USA or 237 High voltage source measure unit, Keithley, USA). Current value at each voltage step was obtained by the customized Labview program. An inverted fluorescence microscope (IX-51, Olympus, Japan) and a CCD camera (DP73, Olympus, Japan) were used to detect and trace ionic plasma image. Commercial software (CellSense, Olympus, Japan) was used to synchronize the CCD camera with the microscope and to analyze the images. Time evolving snapshots of nanoelectrokinetic light-emitting filament were captured using a high-speed camera (Fastcam Mini UX50, Photron, Japan) mainly at 250 fps.

The percent transmittance was measured using U-2900 Spectrophotometer (Hitachi, Japan). First, a transmittance through bare glass was measured as a reference. Then, a transmittance through dry Nafion film on top of slide glass and wet Nafion film (by 2 M NaCl) on top of slide glass were measured. Their ratios were calculated as % transmittance.

## Results and Discussions

### A new concept for direct visualization

**Figure 2:**
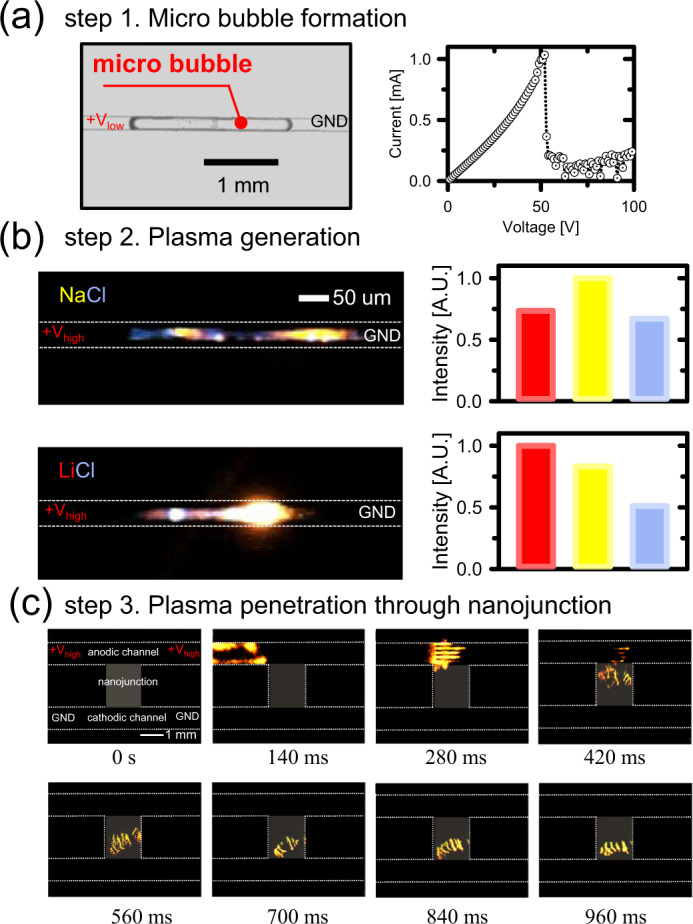
Experimental demonstrations of the visualization. (**a**) Micro bubble formation with electrical current plot as a function of applied voltage. (**b**) Plasma generation using high electric voltage for NaCl and LiCl electrolyte solution. (**c**) Time evolving snapshots of the penetration of the plasma through the nanoscale space.

A schematic illustration of direct visualization was provided in Fig. 1(a). Applying relatively low voltage on anodic channel, a bubble was generated due to the Joule heating as shown in step 1 of Fig. 1(a). High voltage, then, was applied to the anodic channel to initiate the plasma discharge inside the bubble, since high electrical resistance focused the electric potential only to the bubble (step 2 in Fig. 1(a)). Plasma, so-called the fourth fundamental state of matter, can be generated by subjecting a gas to a strong electric field and the gas becomes an ionized gaseous substance. While it may seem like a spark, the electric spark is one form of plasma state such as coronas, glows and arcs, *etc*. A spark occurs when the electric field across a dielectric medium exceeds the dielectric breakdown strength of the medium and then the medium becomes ionized forming a conducting channel of plasma state^52^. Thus, the electrical discharge across the microbubble may be classified as a spark. In this experiment, since either NaCl or LiCl were injected in the microchannel, sodium, lithium and chlorine would be the possible ions turned to be plasma. Finally, the plasma can penetrate through the nanoscale space by electrical grounding the cathodic channel (step 3 in Fig. 1(a)). In order to demonstrate this concept, a polydimethylsiloxane (PDMS) microchip incorporated with Nafion nanoporous membrane was fabricated as shown in Fig. 1(b). PDMS and glass should be the building block of the microchip, because silicon substrate has lower dielectric breakdown voltage than water so that Si-based device is destroyed when high voltage is applied. Simple straight microchannels were parallelly aligned and Nafion nanojunction (the dimension is 2 mm (length) × 1 μm (depth) × 2 mm (width)) was bridging the microchannels to guide plasma transportation. Nafion contains long chained polymer with highly charged functional group so that it acts as perm-selective material because its pore size distributes in the rage of 1–10 nm^43^. This configuration has been investigated for a couple of decades to develop efficient biomolecular preconcentrator^32,50,53–59^ or nanoelectrokinetic desalination/purification platform^5,60–62^. Both microchannels had the dimension of 2,000 mm (length) × 15 μm (depth) × 1 mm or 50 μm (width). The fabrication of PDMS microchannel followed general soft-lithographical method^46^ and Nafion was patterned using surface patterning method^32,47,50,63^. Aforementioned schematics of each step were experimentally demonstrated as shown in Fig. 2.

### Micro bubble formation

Either NaCl (2 M) or LiCl (2 M) was injected into both channel and electrical voltage was applied only through the anodic channel in step 1 using high voltage power supplier (PS 350, Stanford Research Systems, USA or 237 High voltage source measure unit, Keithley, USA) under microscopic observation by inverted microscope (IX53, Olympus, Japan). As the voltage increase, current initially increased linearly but it suddenly dropped after 50 V, indicating an electrical shortage due to a bubble generation (Fig. 2(a)). In the meantime, microscopic observation verified the formation of bubble inside the anodic channel. This was attributed to the Joule heating over a threshold voltage due to the finite current flowing through the microchannel. As shown in Fig. 2(a), the current increased up to 1 mA at 50 V right before the formation of bubble. Thus, the input power was 50 mW. The water volume and mass inside the microchannel were 1.5 nL and 1.5 μg (*i*.*e*.15 μm (depth) × 50 μm (width) × 2 mm (length)), respectively, so that Joule heating can elevate the temperature enough to boil the liquid inside a microchannel. Without consideration of diffusive heat loss through PDMS, the temperature could be elevated to over 8000 °C (*i*.*e*. 50 mJ/Cp/1.5 μg where Cp = 4.2 J/g·°C) in 1 sec. Practically, the temperature cannot rise indefinitely (nowhere close to 8000 °C). This was confirmed by observing the PDMS microchannel is not broken or burned. The composition of bubble will be mainly water vapor. Note that the bubble formed can also have oxygen, carbon dioxide release from water (previously dissolved in spontaneous process). Thus, if one need to develop this platform for sensing unknow ion targets, one need to have careful degassing process before the formation of bubble. However, the degassing is unnecessary because the main object of this work is the visualization of rejection of chlorine ions through nanoscale space.

While the bubble was generated at random position, the higher electrical resistance of the microchannel than that of the reservoir will prefer the bubble formation in the middle of microchannel. See Supporting Video 1 for the micro bubble formation. The bubble formation experiments were repeated more than 30 times which has enough number to guarantee the statistical meaning. If it is performed in larger scale (for example, volume of 1 cm × 1 cm × 1 cm), less than 1 °C can increase so that it is hard to form a bubble. Also, only one bubble was formed since the electric field was focused to the bubble once the bubble was generated. This focusing of the electric field within the bubble is not relevant to the bubble formation itself. It becomes important for the next step, *i*.*e*., the electrical breakdown of the bubble. Note that the electric current was still measurable after forming the bubble mainly because remaining thin liquid film on the surface of PDMS still conducted the current.

### Plasma generation

Switching the applied electrical voltage over 500 V enabled to discharge a plasma inside the micro bubble (Fig. 2(b)). The transport of ions at the interface between liquid and plasma is an active area of research in the sub-discipline of plasma physics studying the atmospheric pressure electrical discharges or discharges in liquid. It is experimentally well known that the interfacial transportation of solutes from liquid is extremely enhanced when the contacting gas becomes plasma state^40,64–68^. As an example, Shirai *et al*. suggested a localized boiling of water interface in contact with the plasma is responsible for the enhanced transport of sodium atoms^40^. At the electrical breakdown of the microbubble (*i*.*e*. generation of plasma), therefore, ions (sodium, lithium and chlorine) can be transported into plasma entrained in the boiling vapor from the electrolyte.

Since the vapor inside the bubble contained chlorine atoms in common and either lithium or sodium atoms, unique flame colors were emitted from the plasma. (Representing color of each atoms were violet, red and yellow for chlorine, lithium and sodium, respectively^69^) Especially, chlorine atoms entrained in the plasma can contribute to the violet color of the emission assuming that microbubble contains no solute atoms or ions other than sodium and chlorine^45^. The presence of sodium atoms is more easily identified by the characteristic strong yellow emission at 589 nm. Microscopic images in Fig. 2(b) were captured even without background light because of a strong emission from the plasma itself. See Supporting Video 2 for each electrolyte solution. Image analysis (ImageJ, NIH and Photo Shop, Adobe) confirmed that the plasma emitted all of three colors inside the bubble. Black background and saturated area were subtracted from original image and counting the number of pixels for three color bands. Then, the values were normalized with the maximum value among them. Note that conventional photo-spectrometer was unable to measure the color spectrum due to fast and fluctuating plasma generation^38,39^. While red and yellow were mixed in both cases of NaCl and LiCl, the important observation was that the violet, which is unique emission of chlorine plasma, was comparable to other colors.

### Plasma penetration through nanojunction

In the meantime, the plasma filament slowly moved toward Nafion nanojunction and penetrated the nanojunction by electrical grounding the cathodic microchannel in step 3. Since plasma and Nafion are conductive matter, electroosmotic flow pushed the bubble toward the nanojunction^70^. Time evolving snapshots were given in Fig. 2(c) using a high-speed camera (Fastcam Mini UX50, Photron, Japan). See Supporting Video 3 for the penetration light-emitting filament through nanojunction.

**Figure 3:**
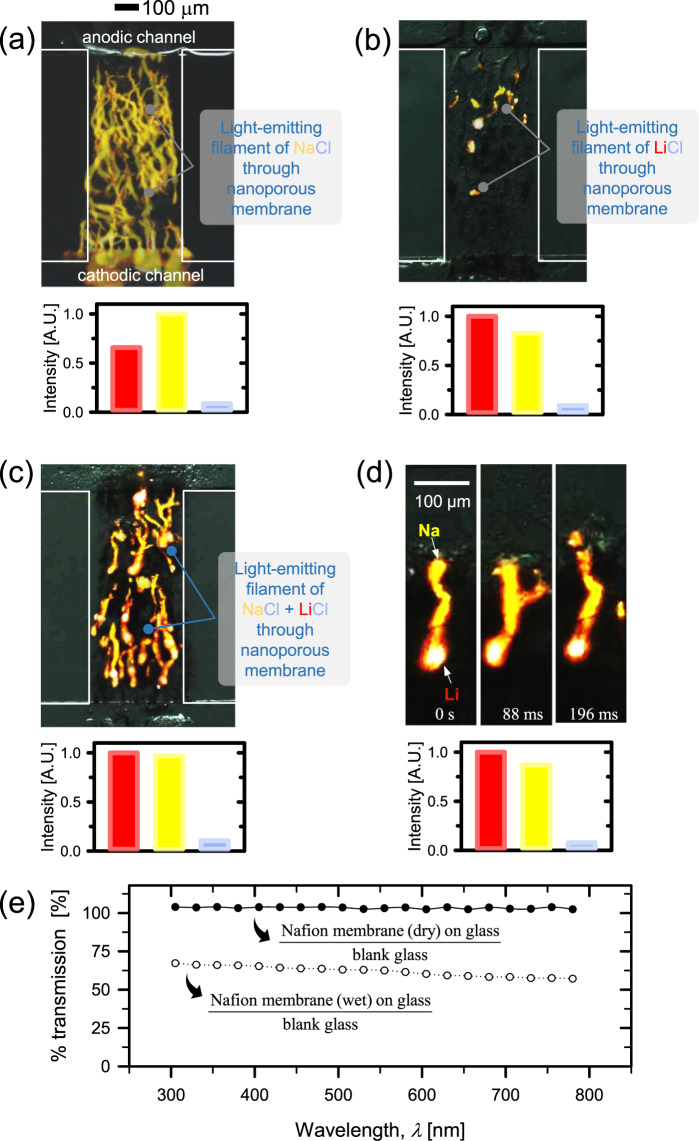
Overlaid images of plasma penetration in the nanojunction for (**a**) NaCl, (**b**) LiCl and (**c**) the mixture of NaCl and LiCl. (**d**) Magnified snapshot of one strip of plasma to identify the faster migration of lithium plasma than that of sodium plasma. (**e**) Transmission through Nafion membrane as a function of wavelength. These results indicated Nafion is transparent at most of visible light.

The featured observation in this work was that the color of plasma during the penetration mostly excluded the violet emission from chlorine atoms in both cases of NaCl (Fig. 3(a)) and LiCl (Fig. 3(b)). This reflected that anionic species was rejected by cation selective nanojunction. Since plasma retains an ionic gaseous state, the plasma of chlorine atoms still possesses an anionic characteristic. Whereas yellowish and reddish plasma can pass through the nanojunction, we can visually confirm the cation selective ion transportation.

While chlorine ions preferentially exist in liquid state, chlorine atoms, chlorine ions and Cl_2_ can co-exist in the plasma state. The number density of chlorine atoms can be much higher than or comparable to that of Cl_2_ in the plasma state^71^. The excited neutral Cl (mainly via electron impact dissociative excitation) atoms emit the violet color, which is why the plasma emits violet color for the cases of NaCl or LiCl solutions. As previously stated, the strongly electronegative chlorine atom can actively capture an electron inside the Debye sheath layer (*c*.*f*. Electronegativity of chlorine atom is much higher than F and SO_3_ of Nafion^72^) and chlorine atom turns into chlorine ion. This is why violet emissions are absent in and after the nano space. Similar scenario can be applied to sodium and lithium so that they can pass through the nano space.

Note that 250 of high-speed images were overlaid in Fig. 3(a,b). While clear transportations were captured in the case of NaCl (Fig. 3(a)), only few (or short) of light-emitting filament was observed in LiCl (Fig. 3(b)). The speed of propagation of lithium is much faster than that of sodium so that lithium plasma filament is shorter than sodium plasma filament because the imaging exposure time is the same. This was attributed to faster mobility of lithium ions than one of sodium ions. In aqueous state, the mobility of lithium ions is smaller than one of sodium ions due to hydrated shell of water molecules^70,73^. Therefore, lithium ion can flow faster than sodium ion if shells can be stripped off. This can be done by strong electric field^74^ or confined nanostructures^73^. Here, lithium ion can move without such heavy shells in plasma state so that it transported faster than sodium ion. Thus, only fewer light-emitting filament was captured at the same frame rate of high-speed imaging.

This faster migration of lithium than sodium was directly observable with the mixture of NaCl and LiCl solution. Both electrolytes were injected into the anodic microchannel and the same experimental procedures were repeated. As shown in Fig. 3(c), the plasma of both sodium atoms and lithium atoms with minimum violet emission was passing through the nanoscale space. One strip of plasma was magnified in Fig. 3(d). As directly visualized here, reddish plasma always leads ahead yellowish plasma. This observation clearly demonstrated faster mobility of lithium ion than one of sodium ion when there were no hydrated shells.

To verify the absence of violet spectrum in Nafion nanojunction, the transmittance of Nafion for visible lights was measured. In Fig. 3(e), % transmission (%T) is the transmission ratios of target specimen to blank slide glass (1 mm thick) so that, for example, 50% indicated target specimen absorbed half of emission at the specific wavelength. As shown, %T is almost 100% in all of wavelength range (300 nm~800 nm) for thin and dry Nafion film on top of glass. Furthermore, thin and wet (by 2 M NaCl solution) Nafion coated on top of glass provided nearly 60% of %T in all of wavelength range. These results confirmed that Nafion is transparent at all of visible light including violet emission. This result can be also confirmed by a previous literature^75^, suggesting that absorbance can be degraded if Nafion is exposed at high temperature over 100 °C (which is not applicable in our experiment) or aged more than a month (also not applicable in our experiment). Note that the transmittance was 60% in the case of wet Nafion because swollen Nafion scattered an excitation source. The swollen Nafion layer develops irregularities on its surface without proper sealing. In our experiment, however, Nafion was not swollen by sandwiching between PDMS-glass bonding.

## Conclusions

Here we experimentally demonstrated a clear evidence of perm-selective ion transportation through the nanoscale space (or nanochannel) by the direct visualization using a microfluidic plasma generation. Micro/nanofluidic platform was employed to generate an ionic plasma inside a microchannel. By pushing the plasma into the nanojunction, light emissions only from cationic species penetrated through the nanoscale space, while one from anionic species was rejected from entering the nanojunction. It, for the first time, visually and directly confirmed the *in situ* perm-selective ion transportation. More importantly, the plasma of lithium, which has lower mobility than sodium in aqueous state, passed the nanojunction faster than sodium presumably because the hydrated shells around lithium would be stripped out in the plasma state.

Since one can obtain the plasma within a gas bubble (diameter ~ O(10) μm), the spatial resolution of conventional microscope is enough to detect the color. For the temporal resolution, all of Fig. 3 were captured with high speed camera at 250 fps. Recently, most of cell phone camera provides such fps so that one can develop the detector with a conventional cell phone. However, the most critical issue is wavelength resolution. While one can just see the color of plasma, rigorous detection of ions should be done by sweep the detection frequency of color emission with expensive and sophisticate equipment such PMT. Therefore, the concept in this work should be used as a pre-screening test, not a sensitive quantification. For identifying the element more precisely, we would suggest high-resolution spectroscopic measurements and rigorous analysis of the emission spectra.

Still, this visualization technique would be a simple but an effective mean not only for characterizing the ion-selectivity of nanostructures but also for answering fundamental questions arisen in nanoscale electrokinetic and plasma physics research field. Note that we also have tried with potassium, which has greenish emission, but its emission was too weak to be detected. In engineering aspect, however, one could utilize this technique to detect unknown cationic species from certain sample fluid with minimum sample volume, if one can afford photo-analysis tools of high-resolution. In such applications, the sample liquid should be carefully degassed for removing O_2_ and spontaneously dissolved CO_2_ to prevent false detection.

## Supplementary information

Supplementary Video 1.

Supplementary Video 2.

Supplementary information.
